# Bridging Museum Mission to Visitors’ Experience: Activity, Meanings, Interactions, Technology

**DOI:** 10.3389/fpsyg.2019.02092

**Published:** 2019-09-10

**Authors:** Annamaria Recupero, Alessandra Talamo, Stefano Triberti, Camilla Modesti

**Affiliations:** ^1^Department of Social and Developmental Psychology, Faculty of Medicine and Psychology, Sapienza University of Rome, Rome, Italy; ^2^Department of Oncology and Hemato-Oncology, University of Milan, Milan, Italy; ^3^Applied Research Division for Cognitive and Psychological Science, European Institute of Oncology (IEO) IRCCS, Milan, Italy

**Keywords:** museum, visitor experience, augmented reality, virtual reality, activity theory

## Abstract

In recent years, the contribution of various disciplines and professionals (i.e., from marketing, computer science, psychology, and pedagogy) to museum management has encouraged the development of a new conception of museology. Specifically, psychology has affected the overall conception of museum and the visitors toward a more holistic vision of the museum experience as a complexity of memory, personal drives, group identity, meaning-making process, as well as leisure preferences. In this regard, psychological research contributes to advance the scientific knowledge about psychological and social phenomena related to the visitor experience, as well as to design innovative technologies and future tourism services. The present contribution discusses the Socio-Cultural Activity Theory (AT) as a theoretical framework to conceptualize the museum visit as an activity mediated by the technology, and to better identify the factors shaping the interaction between the visitors and the technologies. To do so, a case study is presented: a qualitative research performed at the Ara Pacis Museum in Rome (Italy) to analyze the visitor experience of a tour that integrates augmented and virtual reality. Information derived from applying AT on visitors’ experience highlight the value of technology as mediating tool between the museum mission and the visitor experience, considering the interaction between visitors’ characteristics, museum environmental dimensions, and technology’s features.

## Museum as Evolving Cultural Environments

In the last years, museums are becoming more audience-centered and such trend requires to consider the needs of the audience (the actual as well as the potential visitors) when planning activities and exhibitions (Hooper-Greenhill, 2006). Furthermore, museums need to adapt themselves to the socio-cultural changes, by finding new ways to meet the emerging demands related to learning and enjoyment (Black, 2005).

Such evolution is especially pushed by social, political, and economic pressures (Ross, 2004; Bearman and Geber, 2008; Black, 2012). Ballantyne and Uzzell (2011) explain the audience-centered approach as a strategy that museums adopt in response to the decline in public funding, as well as to compete with other leisure services in engaging consumers.

In addition to the external pressure, there was a change in perspective based on new epistemology for conceiving museums and the contamination with different disciplines, especially psychology and the social sciences.

Such profound change is conceptualized twofold, concerning both the shift from the old museology based on the positivism–behaviorist approach to the new museology based on a constructivist approach, and a change from a collection-centered to an audience-centered focus: while the traditional conception focused on the exposition and tended to direct visitors’ behavior throughout pre-determined paths, museum managers today are prone to explore visitors’ perspective in order to collaboratively build meaningful experiences (Hooper-Greenhill, 2000; Simon, 2010). In addition to learning outcomes, museums value emotions, fun, and meaning making (Hooper-Greenhill, 2004).

In light of the above, an increasing number of museums are seeking innovative solutions to better exhibit and communicate the tangible and intangible heritage they preserve, while engaging visitors in an educational yet leisure experience.

Nowadays, the most promising technologies are those laying in the category of computer-mediated reality. Augmented reality (AR) refers to a system that integrates digital contents (i.e., texts, images, videos, and 3D objects) with the user’s perception of the real world, through the use of smart glasses, tablet, and smartphone. It provides an environment where users can view a combination of virtual and real objects, while the virtual reality (VR) immerses the user into a virtual environment (Phon et al., 2014).

With regard to the cultural heritage, such technologies are applied especially in archeological and historical museums, in order to support visitors in imagining how sites or monuments could originally have looked like (Vlahakis et al., 2001; Tzortzaki, 2002; Damala et al., 2007; Cultraro et al., 2009; Petrucco and Agostini, 2016). Indeed, AR and VR allow to expose users to stimuli and complex situations that are normally impossible to reproduce in the museum physical location. Moreover, AR and VR are characterized by inherently engaging properties (e.g., users often perceive them as interesting, funny, intriguing, and may prefer them over more traditional devices), and they are scalable and adaptable to different contexts and issues, in that virtual environments and digital stimuli can be designed *ad hoc* (Recupero et al., 2018).

Besides the amount of literature on this topic, there are still some open issues that need to be addressed so to better understand how technological innovation supports museum mission and enhance the visitor experience. Moreover, in order to design innovative solutions, the mediation of the technology needs to be investigated considering the interaction between visitors (i.e., their previous experience, interest, and motivation), technology (i.e., its features and mode of interaction), and museum context (i.e., mission and strategies, physical environment, and display of the artifacts).

In this regard, the relationship between the tourism sector and the psychological research is bidirectional: in the attempt to improve the offer, museums pose considerable research challenges which can be addressed by adopting psychological frameworks and methods. In this way, psychological perspective is experimented in an emerging field of study to derive relevant findings and guide the future tourism services design.

The present contribution explores this process by discussing the Socio-Cultural Activity Theory (AT) as a theoretical framework to conceptualize the museum visit as an activity mediated by the technology, and to better identify the factors shaping the interaction between the visitors and the technologies. To do so, a case study is presented: a qualitative research performed at the Ara Pacis Museum in Rome (Italy) to analyze the visitor experience of a tour that integrates augmented and VR.

## How Psychology Contributes to Museum Visitor Studies

In recent years, the contribution of various disciplines and professionals (i.e., from marketing, computer science, psychology, and pedagogy) makes “Visitor Studies” a growing research field with the aim of creating a more systematic field study on museum visitors (Goulding, 2000; Kirchberg and Tröndle, 2012), considering the visitor experience as a complexity of memory, personal drives, group identity, decision-making, and meaning-making strategies, as well as leisure preferences (Benefield et al., 1993; Bitgood, 2002; Falk and Dierking, 2016).

For example, environmental psychology’s perspective is used to analyze museums as a source of emotions and affect (Cancellieri et al., 2018). Indeed, there is a significant relationship between the museum’s perceived quality and elicited emotions, which in turn affects visitors’ satisfaction (De Rojas and Camarero, 2008).

Psychology has also affected the overall conception of museum and the way the visitor experience is conceived and studied. Indeed, in recent years there was a shift from the “old museology” based on the positivism-behaviorist approach, to the “new museology” based on constructivist approach (Vergo, 1997).

The old museology conceived the museum as an institution devoted to the education of the masses (Hein, 2002). The model of communication expert-to-novice placed the museum curator in charge of defining the information to be transmitted, while the visitors were expected to absorb and retain the message (Macdonald, 2006). The museum collection was the focus of attention, in order to find the most effective way to convey the inherent meaning of the exhibited artifacts (Hooper-Greenhill, 2006).

By introducing the “New Museology,” Vergo (1997) stressed the need for a re-examination of the role of the museum and its relationship with the visitors and the society.

The shift from the old to the new museology was part of a broader development in many social disciplines that took place during the 1980s and 1990s. Specifically, the diffusion of the Constructivism (with the fundamental contributes of Kelly, Bruner, Mead, Piaget, Lewin, Vygotskij) fostered “reflexivity” among the museum community – in the form of greater attention to the processes by which knowledge is produced and to the partial nature of knowledge itself (Macdonald, 2006).

Thus, in the last 20 years a debate about the social and political roles of museums has been raised, encouraging new forms of communicating the cultural heritage in contrast to the collection-centered approach (McCall and Gray, 2014).

In such perspective, the museum is conceived as a “facilitator,” enabler, and mediator of learning since it provides the suitable setting to foster the meaning making of visitors, according to their specific background and needs (Hooper-Greenhill, 2006; Black, 2012).

### The Contribution of Activity Theory

A further step toward a comprehensive understanding of visitor experience derives from the analysis of visitor interaction with technology used as interpretative tool. To this end, the research can benefit from the Cultural–Historical AT (Leont’ev, 1974, 1978; Engeström, 1987, 2000) as framework to guide both the research on and the design of interactive technologies.

Starting from Susan Bødker (1987, 1990) works, AT was getting the attention of human–computer interaction (HCI) community in line with the attempt to go beyond the cognitive paradigm. Indeed, it was recognized that users’ behavior cannot be explained only as a function of their individual mental activity, and the interaction with the technology is not a matter of input–output exchanges between information-processing units (Norman, 1980; Bannon, 1991, 2011).

Nowadays, there is evidence that it is important to consider ecological criteria for designing technologies (Talamo et al., 2011a, b; Giorgi et al., 2013), avoiding an idealized and rational model of the practices that inevitably fails to capture the complexity and contingency of real-life actions in specific situations (Talamo et al., 2015).

Among psychological theories, AT has been recognized as a powerful framework since it explores the interaction between users and tools, and thus it can be successfully applied to study the technology in the context of human activities (Kaptelinin and Nardi, 2006).

Activity Theory conceptualizes the human activity as a form of doing performed by a subject and directed to an object that is a prospective outcome that meets certain needs of the subject. This interaction between the subject and the object can be mediated by the tool, a physical artifact or an intangible tool (e.g., ideas and procedures) that allows the subject to reach the object (Leont’ev, 1974, 1978).

The tool as a mediator of the activity can be both enabling and constraining: it empowers the subject by enabling him/her to reach the outcome, but it also restricts the interaction to be from the perspective of that particular tool (Kuutti, 1996). As a mediating tool, the technology may enable an activity that cannot be practically possible and feasible, or it may enable an activity to have an object that would otherwise been impossible to grasp (Kaptelinin and Nardi, 2006).

Activity Theory, by focusing on person’s intentionality, tool mediation, and activity analysis, allows museum researchers and managers to consider how the technology impact on visitors’ experience in the situated context of exhibitions.

## Case Study

The case study described hereafter aims at highlighting how AT could be used as lens for the analysis of museum visitor experience mediated by AR–VR technology.

The research context is the Ara Pacis Museum in Rome (Italy) built around the monument of the “Ara Pacis Augustae,” an altar dedicated to the Roman goddess of Peace that served as a mean to celebrate a long period of peacefulness, abundance, and prosperity approximately from 27 B.C. to 180 A.D.^1^

The monument consists of an open-air altar surrounded by precinct walls sculpted entirely in Luna marble. It is exhibited within the architectural structure designed by Richard Meier & Partners Architects.

From 2016, the museum offers the tour “Ara as it was” that combines AR and VR, telling the story of the monument and immersing the visitor into the original context of the Ara Pacis.

To perform the tour, the visitors wear Samsung Gear VR headset that includes the visor combined with the Samsung Galaxy S7 smartphone and the headphones.

The tour is organized into nine points of interest (POIs). The first two POIs are based on VR: the visitors, seated on the chairs, are greeted with a 360° filmed view of the Ara Pacis in the Campo Marzio (the original location of the monument) and then virtually attend the sacrificial ritual performed by actors within a virtual scenario at the time of Ancient Rome. The following seven POIs are located around the monument, where the view of the altar’s details is augmented with the colors and the related audio description.

### Research Objective and Methodology

The research is an ethnographic investigation aiming at describing the experience of visitors who performed the Ara as it was tour, so to reflect on the added value that technologies provide to the museum.

Some studies have already analyzed the user experience with AR and VR in cultural heritage contexts, in order to guide the future design of innovative technologies. For example, Jung et al. (2016) investigated the impact of AR and VR on the museum visitor experience, by applying social presence theory and considering the four realms of experience economy (entertainment, education, esthetic, and escape experience). tom Dieck et al. (2016) performed a qualitative research to derive design requirements for AR applications in art gallery. From interviews with participants elaborated through an affinity diagram, the authors derived a list of recommendations beyond just easiness and quality of the contents, including comfort, novelty, and hedonic attributes. The study by Petrucco and Agostini (2016) is focused on the learning process stimulated by the use of AR–VR application to discover the Roman remains in Verona. The research employed observation and interviews with pupils and teachers to analyze the impact of the technology on formal and informal learning process.

While most of the literature is focused on the experience of visitors, there is the need to consider the peculiarities of the museum where the technology is implemented. As in the study of Damala and Stojanovic (2012), technological innovation should be considered in the light of the strategies the museum adopts to promote the heritage and to engage the audience of regular and potential visitors.

Besides the amount of literature on this topic, there are still some open issues that need to be addressed so to better understand the interaction between visitors (i.e., their previous experience, interest, and motivation), technology (i.e., its features and mode of interaction), and museum context (i.e., mission and strategies, physical environment, and display of the artifacts).

To address this challenge, the research has been driven by the following research questions (RQs):

• What are the motivations that drive visitors to visit museums using AR–VR technologies?
• What are the outcomes resulting from the visit experience?
• How does the technology mediate the museum visit experience?

The research was performed in compliance with the code of ethics of psychology professionals. The research has been approved by the Ph.D. Council of the Department of Social and Developmental Psychology of the Sapienza University of Rome.

#### Data Collection and Analysis

Ethnography in the domain of Museum Visitor Studies is widely used, in line with the constructivist and socio-cultural turn of the new museology. Similarly, the adoption of ethnography in the field of HCI derives from the need for a holistic perspective and qualitative methods to deeply investigate work and every day practices as they occur in the natural context.

Data were collected through a mixed method in order to focus the attention on the personal, environmental, and technological components of the experience, by integrating the auto-ethnography, observation, and the interviews. Such mixed method improves the richness of the data and provides a comprehensive dataset to analyze the complexity of the user experience (Preece et al., 2002).

The auto-ethnography (also called self-ethnography or reflexive ethnography) involves researcher’s self-observation and reflexive investigation during the exploration of the research context (Dourish, 2006; Hammersley and Atkinson, 2007; Maréchal, 2010; Draper, 2015).

At the Ara Pacis Museum, two sessions of auto-ethnography were performed in order to explore the personal visitor experience within the research context by visiting the museum and using the technology. In this way, the museum identity and the properties of the tour were investigated and the research design was further defined.

Instead of searching for objectivity and reducing the source of “bias” that is the researcher’s experience and influence, the aim was to use the researchers’ personal experience as a source of information about both the topics under investigation and the way they are investigated (Maréchal, 2010).

As the main method to collect data on real users, semi-structured narrative interviews were introduced (Atkinson, 1998). The questions asked were mainly open-ended so to foster the respondents to describe their experience and point of view, without forcing them to select from pre-defined answers. Although the interview framework has been defined to cover a list of pre-defined questions, it was flexible enough to be adapted to the narration of the interviewed, so to expand and enrich the answers (Silverman, 2015).

We performed the interviews with the museum curator, the project manager, and the designer in order to investigate what is called the design-for-use (Folcher, 2003) or technology-as-designed (Carroll et al., 2002). Specifically, they were asked to describe the objectives and expected benefits that motivated the introduction of the Ara as it was tour, as well as the design process and the decisions made about the technology and the organization of the tour.

Twenty-one Italian visitors were involved in the research to perform the interviews at the end of the museum visit, in order to collect data about the design-in-use (Folcher, 2003), meaning how they experience the tour using the technology according to their expectations, motivations, emotions, and interaction experience. Table 1 reports data about the visitors involved in the research.

**TABLE 1:** Information about interviewed visitors.

**Sex**	**Number of visitors**
Male	16
Female	5
**Age**	
18–34 years old	9
35–54 years old	7
+55 years old	5
**Component**	
Couple	13
Small group	8

The interview with the visitors covered the following questions to stimulate the narration about the visit experience:

- Have you ever used AR and/or VR technologies before?
- Why did you decided to visit the museum and perform the tour?
- What did you expect from this experience?
- How did you experience the tour?
- What emotions did you feel during the visit?
- What are the benefits you gain from this experience?
- What are the problems and difficulties you met during the visit?

In addition to the interviews with the visitors, we also employed the “shadowing” technique. This is a form of observation that is often used in workplace studies to observe participants as they move inside different contexts (McDonald, 2005; Hammersley and Atkinson, 2007), and also to observe how the artifacts mediate between people and contexts, and create a source of articulation work (Mellini, 2013; Talamo et al., 2015).

Shadowing differs from the stationary observation that is commonly used in Visitor Studies, when the researcher stays in one room/area of the museum and observes the interaction of the visitors with the exhibits (Hein, 1999). Stationary observation is a useful method to evaluate the exhibits (i.e., attracting power and reading of the panels), but it provides only snapshots of the visitor experience. At the contrary, shadowing allows the researcher to observe the whole experience within the museum context for the entire duration of the tour.

Shadowing differs from participant observation because the researcher did not interact with the visitors. Since this method requires to balance researcher’s presence and participant’s committment (Gill et al., 2014), the participation of the researcher to the visit was not employed in order to allow the visitors to enjoy the visit as a typical cultural leisure activity. Explanations about what has been observed were then asked during the interviews at the end of the visit.

During the shadowing, several elements were recorded: path and movements inside the museum space; superficial/deep observation of the monument and artifacts; use of other tools (i.e., panels); and interaction with the devise, comments, and dialogue with others (staff and visitors).

The visitors waiting to start the tour were approached and asked to participate in the research. They were informed about the research objectives, the collection of anonymous data used only for research purposes through the observation of their visit, and the interview about their opinions.

For performing data analysis, all raw data (interview transcripts, field notes from auto-ethnography, and shadowing) were elaborated in the form of the Activity Diagram.

The Activity Diagram is an adaptation of Young’s (2008) Mental Model: an affinity diagram of user’s activities and goals matched with existing tools, services, and products. It is used in the design practice to identify opportunities for designing innovative solutions based on the lack of the existing tools. The Activity Diagram maps users’ activity system related to the museum visit, through the lens of the AT. Specifically, it maps the hierarchical levels of the activities and the mediating tools that are of paramount importance because they enable the visitors to reach their objectives.

**FIGURE 1:**
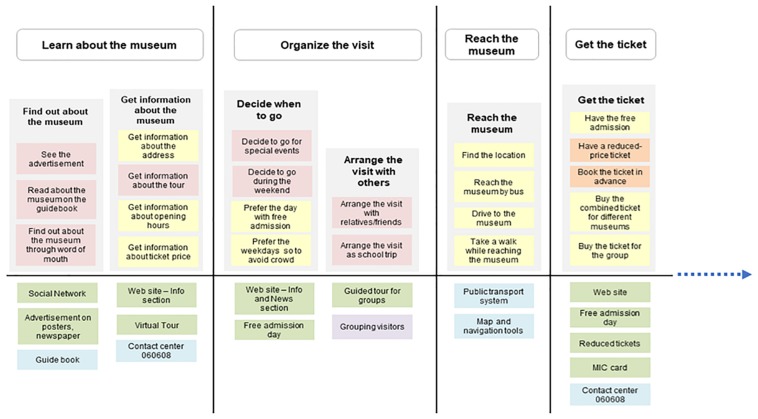
First portion of the Activity Diagram representing: in the upper part, the preparatory activities in the pre- phase of the visit (learn about the museum, organize the visit, reach the museum, and get the ticket); in the bottom part, the existing services/tools/information provided by both the Ara Pacis Museum and other service providers, that the visitors used to perform the above activities.

**FIGURE 2:**
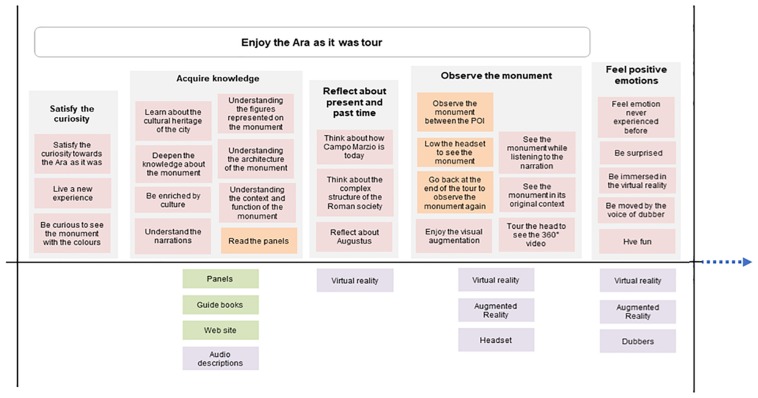
Second portion of the Activity Diagram representing: in the upper part, the core activities of the visit tour (satisfy the curiosity, acquire knowledge, reflect about present and past time, observe the monument, and feel positive emotions); in the bottom part, the features of the Ara as it was tour and the museum artifact ecology that the visitors used to perform the above activities.

**FIGURE 3:**
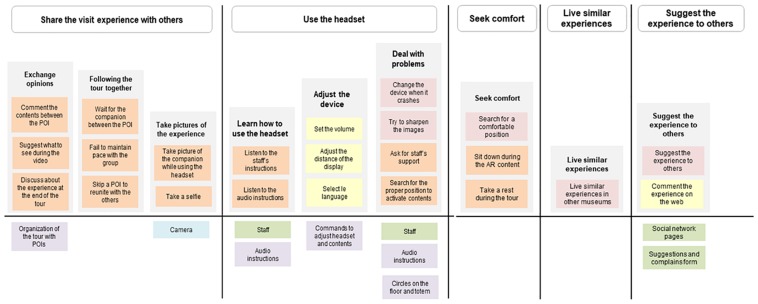
Third portion of the Activity Diagram representing: in the upper part, other core activities of the Ara as it was tour (share the visit with others, use the headset, and seek comfort) and two activities in the post-phase of the visit (live similar experience and suggest the experience to others); in the bottom part, the existing services/tools/information provided by both the Ara Pacis Museum and other service providers, that the visitors used to perform the above activities.

As shown in Figures 1–3, the structure of the diagram includes an upper part with users’ actions grouped based on their goals, and a bottom part with the existing resources that support the related goals.

## Results and Discussion

This paper covers the results gained from the research, in order to explain how the AT points out relevant issues of the visitor experience.

### Technology as a Driver for Museum Visit

Besides the relevant literature about the motivations for visiting museums, the motivations to use AR–VR technology are not specifically addressed, also because the research often involved participants as testers, purposely recruited to assess the technology (see for example, Loscos et al., 2004; Yoon and Wang, 2014).

Visitors decided to visit the museum because they were curious to experience the tour with AR and VR technologies, expecting to enjoy something new and interesting compared to the traditional visit. Specifically, they expected to have a support for imagining the original appearance of the monument and for gaining information about the monument in an engaging way.

Since most of the participants (16 up to 21) have already visited the museum before, technology can be used as a mean for attracting both first-time visitors and repeated visitors by offering an unusual and engaging experience (Black, 2005).

They experienced surprise and fascination when they were projected into the virtual scenario, attending the ceremony with the suspension of disbelief and the illusion of non-mediation (Lombard and Ditton, 1997; IJsselsteijn et al., 2000). Surprise and fascination derived also from the AR effect when the visitors saw the monument turning colors.

With the mediation of the technology, the visit outcomes are not only related to the acquisition of specific knowledge about the monument (i.e., its function, architecture, and decoration), rather they include a general cultural enrichment, pleasure, and enjoyment (Hooper-Greenhill, 2006). Moreover, the visitors reported they enjoy the visit because they share the experience with relatives and friends, having time to exchange opinions and comments between the POIs.

Another relevant outcome reported by the visitors is the desire to live similar experiences in other museums and cultural heritage sites. Thus, the positive experience lived at the Ara Pacis museum improves their interest and constitutes the prior knowledge that reinforces the future intentions (Rennie and Johnston, 2007; Falk and Dierking, 2016; Jung et al., 2016).

Such results about the visit outcomes support the need for considering all the possible and unexpected benefits which range from understanding, value, reflection, meaning making, and the desire to learn more (Hooper-Greenhill, 2006; Black, 2012).

### The Added Value of AR–VR in Visitor Experience

The value of the Ara as it was relies on the possibility it offers to both improve the visual perception of the monument and to immerse the visitors into its original context.

The VR part of the Ara as it was tour provides the experience of immersion and the illusion of being there, in the ancient Campo Marzio attending the ceremony with ancient Roman people.

While the VR is an effective tool to represent the original context and function of the monument, the AR provides the fascinating visualization of the original colors. In particular, the augmentation with the colors enables the visitors to see the monument as it was in its former glory.

Besides the appreciation of the esthetics appearance of the monument, the visual augmentation linked with the audio description allows the visitors to focus the attention on the details and identify the different elements of the reliefs. Specifically, the technology supports the visitors in focusing the attention and interpreting what they observe on the monument, by proposing some key-concepts related to the original socio-cultural context of the monument.

By representing its original appearance and function, the technology acts as a bridge between the visitors’ activity contexts and activity context of the monument (Kaptelinin, 2011).

### Artifacts Ecology

The research was focused on the artifacts ecology (Jung et al., 2008), meaning the multitude of artifacts available in the museum. Indeed, instead of considering the use of a single technology in isolation, research should investigate how people distribute their activities across different technologies, with specific functions or overlapping capabilities (Jung et al., 2008; Bødker and Klokmose, 2012; Bødker, 2015).

The concept of artifacts ecology can be usefully applied to the museum domain, in order to investigate the juxtaposition of different interpretative tools developed over the years and the way visitors used them during the museum experience.

The ecology of the Ara Pacis Museum is composed of the monument, some busts and sculptures which form the museum collection, together with the interpretative tools in the form of scale models of the monument, informative panels, video screen, and the AR–VR technology. Such ecology is the result of a stratification of artifacts over the time: indeed, the introduction of a new technology occurs as an increase of existing tools and practices, and over the years the technological stratification evolves since only rarely a new technology totally replace an existing tool (Bruni and Gherardi, 2007; Talamo et al., 2015).

This is particularly evident in the case of the Ara Pacis Museum, in which the new practice of the Ara as it was tour is embedded in the museum physical contexts with traditional tools like the panels.

During the shadowing, we noted that some visitors read the panels at the end of the tour in order to get further information about the monument and the museum. Thus, the AR–VR technology cannot replace the traditional interpretative tools and it should be properly integrated into the museum context. Specifically, the technology does not replace the scale model of the Ara Pacis with the 360° virtual reconstruction, neither the informative panels with the audio description, since the traditional interpretative tools are used as complementary supports as much as they respond to visitors’ needs and interests (Falk and Dierking, 2016).

As pointed out by Norman (1991) about cognitive artifacts and by Bødker and Klokmose (2012) about artifacts ecology, the introduction of a new tool changes the activity of the users who need to perform additional tasks. Indeed, a relevant portion of the Activity Diagram (Figure 3) is related to the use of the device: learning how to use the headset, adjusting the device, and dealing with problems and malfunctions differentiate the Ara as it was experience from the traditional visit.

Even if the visitors are familiar with AR–VR technologies, additional tasks are requested to understand how to perform the tour, adjust and properly use the headset, and deal with struggles and malfunctions. Thus, high level of usability is necessary, as well as the design of a system of instructions in order to facilitate the visitors in learning how and properly use the technology.

## Conclusion

This paper describes an example of a qualitative research that uses a psychological framework (Socio-Cultural AT) and method (the ethnography) to deal with some emerging challenges of technological innovation in museums. Specifically, the research points out that AR–VR technology can act as a meeting point between the museum’s mission (for exhibiting and communicating the heritage) and visitors’ need for an engaging, educational, and enjoyable experience.

Indeed, museum experience is part of tourists’ hedonic consumption (Skavronskaya et al., 2017) in which learning and leisure are intertwined (Dierking and Falk, 1992; Falk et al., 1998). The unusual or engaging events experienced on a vacation are more likely to elicit positive arousal and are therefore more likely to be remembered (Zhang et al., 2018).

The main findings from the research can be summarized as follows:

• The technology can be exploited as an “attractor,” both for tourists and for local visitors, but it should also act as a “sustainer” during the visit (Edmonds et al., 2006). To this end, the technology should provide a balance between fascination, surprise effect, and quality of the content, so to provide the educational leisure experience the visitors expect to join.
• To stimulate a memorable tourism experience, museums can take advantage from the integration of AR and VR to convey heritage-related information based on the metaphor of the time machine: the augmentation of visitors’ perception with visual and auditory information to discover the intangible dimensions of the tangible heritage, and the immersion into virtual scenarios from the past with the illusion of being there.
• Although such technologies offer promising affordances, they need to be designed according to the peculiarities of the museum context (i.e., the museum artifacts ecology). Indeed, the museum artifacts ecology should properly integrate traditional and innovative interpretative tools, both with diverse and overlapping capabilities, so to meet the need and preferences of diverse visitors.

This study is not exempt from limitations, regarding both the methodology employed and the results gained.

The ethnography as it is used in psychological research represents a valuable tool to analyze museum visit as an activity: indeed, it allows researchers to investigate visitors’ interaction with the technology, going beyond the mere collection of opinions that could be done with structured customer surveys.

Since it is a situated research – performed in a museum with an unique identity, the contents provided during the tour have been designed *ad hoc* for the Ara as it was, and the visitors involved are all Italian adults highly motivated to experience the tour – the results are not intended to be generalized.

Regarding the shadowing, being observed inevitably drive the visitors to adapt their behavior to the research situation. This could be overcome by employing audio and video recording without the need for the presence of the researcher.

Moreover, the visitors involved in the research often visit museums, as a habit during travels and a leisure activity during the free-time, and they are interested in cultural heritage. To further enhance the museum mission, we need to investigate the impact of such new technologies on a different target such as those who never visit as well as those who rarely visit museums. The technology could be successfully exploited to engage this audience, by addressing barriers and negative motivations.

Future research may explore more deeply the subjective experience of visitors, for example to investigate links between the appreciation of technologically mediated museum experiences and specific visit motivations. Furthermore, in order to give museum managers information on which technology possibly implementing in their expositions, future qualitative and quantitative research could compare VR and AR in terms of enjoinment, interest, and experience design.

## Data Availability

The datasets generated for this study are available on request to the corresponding author.

## Ethics Statement

Ethical review and approval was not required for the study on human participants in accordance with the local legislation and institutional requirements. The patients/participants provided their written informed consent to participate in this study.

## Author Contributions

AR drafted the manuscript and wrote the sections of case study and results. AT contributed in conceiving the rationale of the article as a whole, and in writing the first two sections. ST contributed in writing the abstract and conclusions. CM contributed to the manuscript refinement. All the authors provided approval for publication of the manuscript.

## Conflict of Interest Statement

The authors declare that the research was conducted in the absence of any commercial or financial relationships that could be construed as a potential conflict of interest.

## Footnotes

1 For further information about the Ara Pacis Museum see http://www.arapacis.it/en.

## References

[B1] Atkinson R. (1998). *The Life Story Interview.* Thousand Oaks, CA: Sage.

[B2] Ballantyne R. Uzzell D. (2011). Looking back and looking forward: the rise of the visitor-centered museum. *Curator* 54 85–92. 10.1111/j.2151-6952.2010.00071.x

[B3] Bannon L. (1991). “From human factors to human actors. the role of psychology and human-computer interaction studies in systems design,” in *Design at Work: Cooperative Design of Computer Systems*, eds Greenbaum J. Kyng M. (Hillsdale, MI: Lawrence Erlbaum Associates), 25–44.

[B4] Bannon L. (2011). Reimagining HCI: toward a more human-centered perspective. *Interactions* 18 50–57. 10.1145/1978822.1978833

[B5] Bearman D. Geber K. (2008). Transforming cultural heritage institutions through new media. *Mus. Manag. Curatorship* 23 385–399. 10.1080/09647770802517431

[B6] Benefield A. Bitgood S. Shettel H. (1993). Visitor studies: theory, research and practice. *Curator* 36 238–240.

[B7] Bitgood S. C. (2002). “Environmental psychology in museums, zoos and other exhibition centers,” in *Handbook of Environmental Psychology*, eds Bechtel R. B. Churchman A. (New York, NY: Wiley), 461–480.

[B8] Black G. (2005). *The Engaging Museum: Developing Museums for Visitor Involvement.* New York, NY: Routledge.

[B9] Black G. (2012). *Transforming Museums in the Twenty-First Century.* New York, NY: Routledge.

[B10] Bødker S. (1987). Through the interface-A human activity approach to user interface design. *DAIMI Rep. Ser.* 16:161 10.7146/dpb.v16i224.7586

[B11] Bødker S. (1990). Activity theory as a challenge to systems design. *DAIMI Rep. Ser.* 334 10.7146/dpb.v19i334.6564

[B12] Bødker S. (2015). Third-wave HCI, 10 years later - participation and sharing. *Interactions* 22 24–31. 10.1145/2804405

[B13] Bødker S. Klokmose C. N. (2012). “Dynamics in artifact ecologies,” in *Proceedings of the 7th Nordic Conference on Human-Computer Interaction: Making Sense Through Design*, (New York, NY: ACM), 448–457.

[B14] Bruni A. Gherardi S. (2007). *Studiare le pratiche lavorative.* Bologna: Il mulino.

[B15] Cancellieri U. G. Manca S. Laurano F. Molinario E. Talamo A. Recupero A. (2018). Visitors’ satisfaction and perceived affective qualities towards museums: the impact of recreational areas. *Rass. Psicol.* 35 5–18. 10.4458/0135-01

[B16] Carroll J. Howard S. Vetere F. Peck J. Murphy J. (2002). “Just what do the youth of today want? Technology appropriation by young people,” in *Proceedings of the 35th Annual Hawaii International Conference on System Sciences*, (Piscataway, NJ: IEEE), 1777–1785.

[B17] Cultraro M. Gabellone F. Scardozzi G. (2009). “The virtual musealization of archaeological sites: between documentation and communication,” in *Proceedings of the 3rd ISPRS International Workshop 3D-ARCH*, Trento.

[B18] Damala A. Marchal I. Houlier P. (2007). “Merging augmented reality based features in mobile multimedia museum guides,” In *Proceedings of the XXI International Symposium CIPA 2007.* (Athens: CIPA), 259–264.

[B19] Damala A. Stojanovic N. (2012). “Tailoring the adaptive augmented reality (a2r) museum visit: identifying cultural heritage professionals’ motivations and needs,” in *Proceedings of the 2012 International Symposium on Mixed and Augmented Reality (ISMAR-AMH)*, (Piscataway, NJ: IEEE), 71–80.

[B20] De Rojas C. Camarero C. (2008). Visitors’ experience, mood and satisfaction in a heritage context: evidence from an interpretation center. *Tour. Manag.* 29 525–537. 10.1016/j.tourman.2007.06.004

[B21] Dierking L. D. Falk J. H. (1992). Redefining the museum experience: the interactive experience model. *Visit. Stud.* 4 173–176.

[B22] Dourish P. (2006). “Implications for DESIGN,” In *Proceedings of the SIGCHI Conference on Human Factors in Computing Systems* (New York, NY: ACM), 541–550.

[B23] Draper J. (2015). Ethnography: principles, practice and potential. *Nurs. Stand.* 29 36–41. 10.7748/ns.29.36.36.e8937 25942984

[B24] Edmonds E. Muller L. Connell M. (2006). On creative engagement. *Vis. Commun.* 5 307–322. 10.1177/1470357206068461

[B25] Engeström Y. (1987). *Learning by Expanding: An Activity-Theoretical Approach to Developmental Research.* Helsinki: Orienta-Konsultit.

[B26] Engeström Y. (2000). Activity theory as a framework for analyzing and redesigning work. *Ergonomics* 43 960–974. 10.1080/001401300409143 10929830

[B27] Falk J. H. Dierking L. D. (2016). *The Museum Experience Revisited.* New York, NY: Routledge.

[B28] Falk J. H. Moussouri T. Coulson D. (1998). The effect of visitor agendas on museum learning. *Curator* 41 107–120. 10.1111/j.2151-6952.1998.tb00822.x

[B29] Folcher V. (2003). Appropriating artifacts as instruments: when design-for-use meets design-in-use. *Interact. Comput.* 15 647–663. 10.1016/S0953-5438(03)00057-2

[B30] Gill R. Barbour J. Dean M. (2014). Shadowing in/as work: ten recommendations for shadowing fieldwork practice. *Qual. Res. Organ. Manag. Int. J.* 9 69–89. 10.1108/QROM-09-2012-1100

[B31] Giorgi S. Ceriani M. Bottoni P. Talamo A. Ruggiero S. (2013). “Keeping “InTOUCH”: an ongoing co-design project to share memories, skills and demands through an interactive table,” in *Human Factors in Computing and Informatics*, eds Holzinger A. Ziefle M. Hitz M. Debevc M. (Berlin: Springer), 633–640. 10.1007/978-3-642-39062-3_43

[B32] Goulding C. (2000). The museum environment and the visitor experience. *Eur. J. Mark.* 34 261–278. 10.1108/03090560010311849

[B33] Hammersley M. Atkinson P. (2007). *Ethnography: Principles in Practice*, 3rd Edn, Abingdon: Routledge.

[B34] Hein G. E. (1999). “The constructivist museum,” in *The Educational Role of the Museum*, ed. Hooper-Greenhill E. (Milton Park: Psychology Press), 73–79.

[B35] Hein G. E. (2002). *Learning in the MUSEUM.* New York, NY: Routledge.

[B36] Hooper-Greenhill E. (2000). Changing values in the art museum: rethinking communication and learning. *Int. J. Herit. Stud.* 6 9–31. 10.1080/135272500363715

[B37] Hooper-Greenhill E. (2006). “Studying visitors,” in *A Companion to Museum Studies*, ed. Macdonald S. (Oxford, UK: Blackwell Publishing), 362–376. 10.1002/9780470996836.ch22

[B38] Hooper-Greenhill E. (2004). Measuring learning outcomes in museums, archives and libraries: the learning impact research project (LIRP). *Int. J. Herit. Stud.* 10 151–174. 10.1080/13527250410001692877

[B39] IJsselsteijn W. A. de Ridder H. Freeman J. Avons S. E. (2000). “Presence: concept, determinants, and measurement,” in *Proceedings of the Human Vision and Electronic Imaging V*, San Jose, CA.

[B40] Jung H. Stolterman E. Ryan W. Thompson T. Siegel M. (2008). “Toward a framework for ecologies of artifacts: how are digital artifacts interconnected within a personal life?,” in *Proceedings of the 5th Nordic Conference on Human-computer Interaction: Building Bridges*, (New York, NY: ACM), 201–210.

[B41] Jung T. tom Dieck M. C. Lee H. Chung N. (2016). “Effects of virtual reality and augmented reality on visitor experiences in museum,” in *Information and Communication Technologies in Tourism 2016*, eds Inversini A. Schegg R. (Cham: Springer), 621–635. 10.1007/978-3-319-28231-2_45

[B42] Kaptelinin V. (2011). “Designing technological support for meaning making in museum learning: an activity-theoretical framework,” in *Proceedings of the 44th Hawaii International Conference on System Sciences*, (Piscataway, NJ: IEEE), 1–10.

[B43] Kaptelinin V. Nardi B. A. (2006). *Acting with Technology: Activity Theory and Interaction Design.* Cambridge, MA: MIT press.

[B44] Kirchberg V. Tröndle M. (2012). Experiencing exhibitions: a review of studies on visitor experiences in museums. *Curator* 55 435–452. 10.1111/j.2151-6952.2012.00167.x

[B45] Kuutti K. (1996). “Activity theory as a potential framework for human-computer interaction research,” in *Context and Consciousness: Activity Theory and Human-Computer Interaction*, ed. Nardi B. (Boston, MA: MIT Press), 17–44.

[B46] Leont’ev A. (1978). *Activity, Consciousness, and Personality.* Englewood Cliffs, N.J: Prentice-Hall.

[B47] Leont’ev A. N. (1974). The problem of activity in psychology. *Sov. psychol.* 13 4–33. 10.2753/RPO1061-040513024

[B48] Lombard M. Ditton T. (1997). At the heart of it all: the concept of presence. *J. Comput. Mediat. Commun.* 3 107–113. 10.1111/j.1083-6101.1997.tb00072.x

[B49] Loscos C. Tecchia F. Frisoli A. Carrozzino M. Widenfeld H. R. Swapp D. (2004). “The museum of pure form: touching real statues in an immersive virtual museum,” in *Proceedings of the 5th International conference on Virtual Reality, Archaeology and Intelligent Cultural Heritage*, Oudenaarde.

[B50] Macdonald S. (ed.) (2006). *A Companion to Museum Studies.* Oxford, UK: Blackwell Publishing Ltd.

[B51] Maréchal G. (2010). “Autoethnography,” in *Encyclopedia of Case Study Research*, Vol. 2 eds Mills A. J. Durepos G. Wiebe E. (Thousand Oaks, CA: Sage Publications), 43–45.

[B52] McCall V. Gray C. (2014). Museums and the ‘new museology’: theory, practice and organisational change. *Mus. Manag. Curatorship* 29 19–35. 10.1080/09647775.2013.869852

[B53] McDonald S. (2005). Studying actions in context: a qualitative shadowing method for organizational research. *Qual. Res.* 5 455–473. 10.1177/1468794105056923

[B54] Mellini B. (2013). *The Materialization of Articulation Work and its Implications on Nursing Practices.* Ph.D. thesis, Sapienza University, Trento.

[B55] Norman D. A. (1980). Twelve issues for cognitive science. *Cogn. Sci.* 4 1–32. 10.1016/S0364-0213(81)80002-X

[B56] Norman D. A. (1991). “Cognitive artifacts,”In *Designing Interaction: Psychology at the Human-Computer Interface* ed. Carroll J. M. (Cambridge, MA: Cambridge University), 17–38.

[B57] Petrucco C. Agostini D. (2016). Teaching cultural heritage using mobile augmented reality. *J. e-Learn. Knowl. Soc.* 12 115–128.

[B58] Phon D. N. E. Ali M. B. Halim N. D. A. (2014). “Collaborative augmented reality in education: a review,” in *Proceedings of the International Conference on Teaching and Learning in Computing and Engineering (LaTiCE)*, (Piscataway, NJ: IEEE), 78–83.

[B59] Preece J. Rogers Y. Sharp H. (2002). *Interaction Design, Beyond Human-Computer Interaction.* Hoboken, NJ: John Wiley & Sons.

[B60] Recupero A. Triberti S. Modesti C. Talamo A. (2018). Mixed reality for cross-cultural integration: using positive technology to share experiences and promote communication. *Front. Psychol.* 9:1223. 10.3389/fpsyg.2018.01223 30065690 PMC6056812

[B61] Rennie L. J. Johnston D. J. (2007). Visitors’ perceptions of changes in their thinking about science and technology following a visit to science center. *Visit. Stud.* 10 168–177. 10.1080/10645570701585194

[B62] Ross M. (2004). Interpreting the new museology. *Mus. Soc.* 2 84–103. 10.29311/mas.v2i2.43

[B63] Silverman D. (2015). *Interpreting Qualitative Data.* Thousand Oaks, CA: Sage.

[B64] Simon N. (2010). *The Participatory Museum.* Santa Cruz, CA: Museum 2.0.

[B65] Skavronskaya L. Scott N. Moyle B. Le D. Hadinejad A. Zhang R. (2017). Cognitive psychology and tourism research: state of the art. *Tour. Rev.* 72 221–237. 10.1108/TR-03-2017-0041

[B66] Talamo A. Giorgi S. Mellini B. (2011a). “Designing technologies for ageing: is simplicity always a leading criterion?,” in *Proceedings of the 9th ACM SIGCHI Italian Chapter International Conference on Computer-Human Interaction: Facing Complexity*, (New York, NY: ACM), 33–36.

[B67] Talamo A. Mellini B. Giorgi S. (2011b). “Ergonomia sociale,” in *Ergonomia Cognitiva*, ed. Di Nocera F. (Rome: Carrocci), 251–281.

[B68] Talamo A. Mellini B. Ventura S. Recupero A. (2015). “Studying practices to inform design: organizational issues and local artifacts,” in *Designing Technology, Work, Organizations and Vice Versa*, eds Bruni A. Parolin L. L. Schubert C. (Wilmington: Vernon Press), 71–113.

[B69] tom Dieck M. C. Jung T. Han D. I. (2016). Mapping requirements for the wearable smart glasses augmented reality museum application. *J. Hosp. Tour. Technol.* 7 230–253. 10.1108/JHTT-09-2015-0036

[B70] Tzortzaki D. (2002). “Virtual reality as simulation: the cave as “Space of Illusion” in museum displays,” in *Virtual Space*, eds Qvortrup L. Jensen J. F. Kjems E. Lehmann N. Madsen C. (London: Springer), 258–284. 10.1007/978-1-4471-0225-0_12

[B71] Vergo P. (ed.) (1997). *New Museology.* London: Reaktion books.

[B72] Vlahakis V. Karigiannis J. Tsotros M. Gounaris M. Almeida L. Stricker D. (2001). “Archeoguide: first results of an augmented reality, mobile computing system in cultural heritage sites,” in *Proceedings of the International Symposium on Virtual Reality, Archaeology and Cultural Heritage* (New York, NY: ACM), 131–140.

[B73] Yoon S. A. Wang J. (2014). Making the invisible visible in science museums through augmented reality devices. *TechTrends* 58 49–55. 10.1007/s11528-013-0720-7

[B74] Young I. (2008). *Mental Models: Aligning Design Strategy with Human Behavior. Rosenfeld Media.* New York, NY: Rosenfeld Media.

[B75] Zhang H. Wu Y. Buhalis D. (2018). A model of perceived image, memorable tourism experiences and revisit intention. *J. Destinat. Mark. Manag.* 8 326–336. 10.1016/j.jdmm.2017.06.004

